# LncRNA PVT1 as an effective biomarker for cancer diagnosis and detection based on transcriptome data and meta-analysis

**DOI:** 10.18632/oncotarget.20634

**Published:** 2017-09-04

**Authors:** Yunhong Zeng, Tieqiang Wang, Yi Liu, Zhan Su, Pingtao Lu, Xiaoliang Chen, Dongsheng Hu

**Affiliations:** ^1^ Guangming District People’s Hospital of Shenzhen, Shenzhen, Guangdong, P.R. China; ^2^ Shenzhen Guangming District Center for Disease Control and Prevention, Shenzhen, Guangdong, P.R. China; ^3^ Department of Preventive Medicine, Shenzhen University Health Science Center, Shenzhen, Guangdong, P.R. China

**Keywords:** PVT1, carcinoma, metastasis, diagnosis, meta-analysis

## Abstract

**Purpose:**

Long noncoding RNA (lncRNA) PVT1 was detected all types of cancer from Cancer Genome Atlas (TCGA) project; however, the role of PVT1 in cancer is not clear. This study aimed to reanalyze and determine the effect of PVT1 on cancer diagnosis, especially detection in serum.

**Materials and Methods:**

Differential expression of PVT1 between cancers and corresponding normal tissues and receiver operating characteristic (ROC) curve were analyzed for all types of cancers in TCGA database. RevMan5.3, Meta-DiSc1.4 and STATA14.0 were used to estimate pooled diagnostic effects of PVT1 in tissue as well as serum.

**Results:**

Compared to corresponding normal tissues, PVT1 expression was significantly upregulated in 18 types of cancer and further being an effective diagnosis biomarker in 16 of them. For the 23 diagnosis tests performed in tissue, the pooled AUC and diagnostic odd ratio (DOR) were estimated to be 0.81 (95% CI: 0.76–0.86) and 17.25 (95% CI: 8.43–35.27), when the pooled AUC and DOR were 0.83 (95%CI: 0.75–0.91) and 13.86 (95% CI: 4.70–40.66) for serum tests. Furthermore, the pooled sensitivity and specificity were 0.83 (95% CI: 0.76–0.89) and 0.74 (95% CI:0.70–0.84) for tissue as well as 0.81 (95% CI: 0.76–0.86) and 0.76 (95% CI:0.70–0.81) for serum.

**Conclusions:**

PVT1, especially in serum, might be a usable biomarker for cancer diagnosis / detection.

## INTRODUCTION

Long noncoding RNAs (lncRNAs) are the RNA molecules with size exceeding 200 nts and apparently lack of protein-coding capacity [1]. Nevertheless, lncRNAs have been found being involved in almost all aspects of gene expression through interactions with other components such as proteins, RNAs and DNAs [2, 3]. Increasing evidence suggests lncRNAs could be the key regulators of different cellular processes. Moreover, the dysregulation of homeostatic control of lncRNAs biogenesis could be associated with multiple pathological cancers [4, 5]. The regulating lncRNAs have been shown aberrant expression in tumor tissues and participate in the onset of cancer [6–8]. Because of involvement in many cellular caner pathways, abundant lncRNAs were identified by high-throughput RNA sequencing (RNA-Seq), especially in data of the Cancer Genome Atlas (TCGA) project [5, 9], and expected to play crucial role in cancer diagnosis, detection and therapy.

Recently, a long intergenic noncoding RNA PVT1, homologous to the mouse plasmacytoma variant translocation gene (Pvt1), has attracted widespread attention. The lncRNA PVT1 lies in human chromosome 8q24.21, which is a recognized cancer risk locus with the top target of copy number alterations [10], and has been reported to be dysregulated in various human tumors, such as gastric cancer, non-small cell lung cancer, colorectal cancer, esophageal cancer, pancreatic cancer, hepatocellular carcinoma [5, 11, 12]. Abnormal expression of PVT1 in cancerous tissue has confirmed it as an important player in tumorigenesis of cancers [10, 13]. Furthermore, high expression of PVT1 was identified being associated with poor prognosis of patients [14–17]. More importantly, PVT1 could be steadily detected in patient’s body fluid including blood and saliva, and might be a noninvasive biomarker for cancer diagnosis and detection [18–22]. However, the diagnosis effect of PVT1 is not clear in most cancers, while the expression of PVT1 has been tested in 33 type cancers of TCGA database [12]. Moreover, the reported effect of PVT1 on diagnosis and detection is controversial, and no meta-anlysis has investigated the relationship between PVT1 epression and cancer diagnosis and detection.

The present study aimed to analyze the differential expression of PVT1 between types of cancer and corresponding normal tissue, explore the effect of PVT1 on cancer diagnosis with TCGA data, and further pool the cancer diagnosis and detection effect of PVT1 by meta-analysis.

## RESULTS

### The expression of PVT1 in TCGA cancers

**Figure 1:**
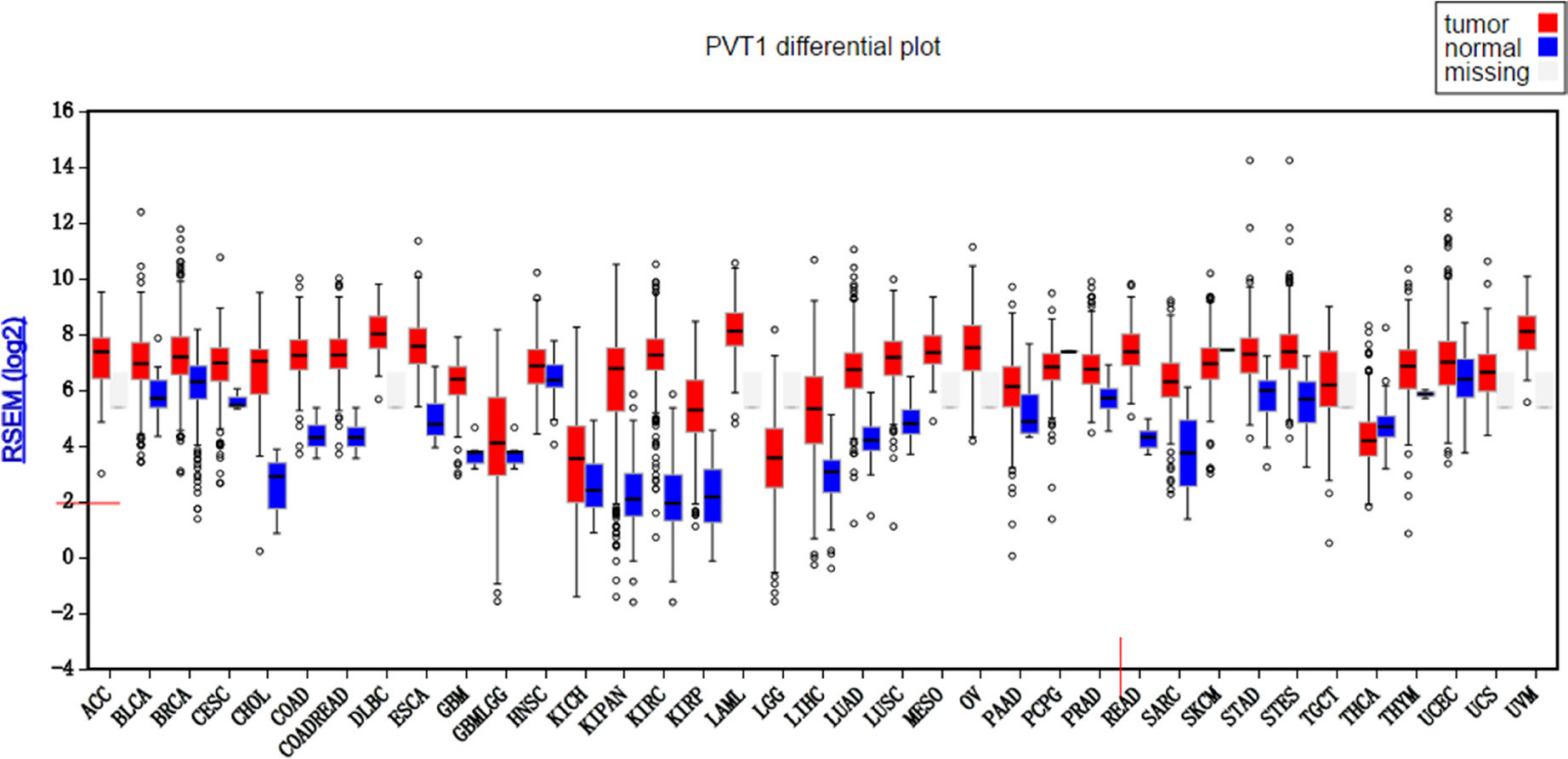
PVT1 differential plot for 32 types of cancers in TCGA database

The expression of PVT1 was checked in TCGA database by firebrowse (http://firebrowse.org/), and PVT1 was stably detected in 32 types of cancer as well as 22 types of corresponding normal tissue (Figure 1). Because of PVT1 detected in only two normal tissues of thymoma patients, PVT1 sequencing data of 21 types were chose to analyze the differential expression between cancers and corresponding normal tissues. The PVT1 expression was significantly upregulated in 18 types of cancers; although, the PVT1 expression of thyroid carcinoma was significantly lower than that of normal tissues (Table 1).

**Table 1:** The differentiation and diagnosis value of PVT1 expression in cancers and corresponding normal ones of TCGA database

Type of cancer	Type of tissue	NO. of Samples	Mann-Whitney U test		Diagnosis tese		
			*Z* value	*P* Value	AUC (95% CI)	Sensitivity	Specificity
BLCA	cancer	407	−4.502	< 0.0001	0.805 (0.718–0.893)	0.801	0.737
	normal	19					
BRCA	cancer	1097	−9.564	< 0.0001	0.785 (0.723–0.806)	0.734	0.663
	normal	121					
CESC	cancer	303	−2.099	0.036	0.774 (0.540–1.000)	0.828	0.800
	normal	5					
CHOL	cancer	36	−4.313	< 0.0001	0.969 (0.915–1.000)	0.972	0.889
	normal	9					
COAD	cancer	286	−93789	< 0.0001	0.963 (0.921–1.000)	0.976	0.953
	normal	43					
ESCA	cancer	184	−5.116	< 0.0001	0.941 (0.879–1.000)	0.859	0.833
	normal	12					
GBM	cancer	154	−3.257	0.001	0.735 (0.602–0.868)	0.708	0.722
	normal	18					
HNSC	cancer	520	−3.204	0.001	0.642 (0.571–0.714)	0.675	0.619
	normal	46					
KIRC	cancer	533	−13.540	< 0.0001	0.988 (0.978–0.998)	0.974	0.959
	normal	73					
KICH	cancer	66	−2.321	0.020	0.658 (0.547–0.769)	0.652	0.720
	normal	25					
KIRP	cancer	290	−8.331	< 0.0001	0.943 (0.910–0.976)	0.886	0.909
	normal	33					
LIHC	cancer	371	−8.619	< 0.0001	0.869 (0.832–0.905)	0.819	0.808
	normal	52					
LUAD	cancer	515	−11.202	< 0.0001	0.938 (0.899–0.978)	0.899	0.885
	normal	61					
LUSC	cancer	502	−10.922	< 0.0001	0.964 (0.947–0.981)	0.924	0.902
	normal	51					
PAAD	cancer	178	−0.847	0.397	0.611 (0.324–0.899)	0.775	0.600
	normal	5					
PRAD	cancer	497	−8.486	< 0.0001	0.854 (0.808–0.900)	0.801	0.755
	normal	53					
READ	cancer	94	−5.253	< 0.0001	0.985 (0.957–1.000)	0.989	0.909
	normal	11					
SARC	cancer	259	−0.550	0.583	0.566 (0.333–0.798)	0.598	0.667
	normal	6					
STAD	cancer	415	−7.541	< 0.0001	0.884 (0.837–0.930)	0.824	0.800
	normal	35					
THCA	cancer	505	−4.589	< 0.0001	0.328 (0.269–0.386)	0.349	0.448
	normal	67					
UCEC	cancer	176	−1.922	0.055	0.619 (0.510–0.727)	0.574	0.600
	normal	25					

Abbreviations: BLCA, bladder cancer; BRCA, breast cancer; CESC, cervical cancer; CHOL, bile duct cancer; COAD, colon cancer; ESCA, esophageal cancer; GBM, glioblastoma; HNSC, head and neck cancer; KIRC, kidney clear cell carcinoma; KICH, kidney chromophobe; KIRP, kidney papillary cell carcinoma; LIHC, liver cancer; LUAD, lung adenocarcinoma; LUSC, lung squamous cell carcinoma; PAAD, pancreatic cancer; PRAD, prostate cancer; READ, rectal cancer; SARC, sarcoma; STAD, stomach cancer; THCA, thyroid cancer.

### Diagnosis value of PVT1 in TCGA cancers

Receiver operating characteristic (ROC) curve was further used to analyze the diagnostic effect of PVT1 in the 21 type cancers. As shown in Table 1, the values of area under receiver operating characteristic curve (AUC) were more than 0.50 in 16 of them, which were 0.805 (*P* < 0.0001), 0.785 (*P* < 0.0001), 0.774 (*P* = 0.036), 0.969 (*P* < 0.0001), 0.963 (*P* < 0.0001), 0.941 (*P* < 0.0001), 0.735 (*P* = 0.001), 0.642 (*P* = 0.001), 0.988 (*P* < 0.0001), 0.658 (*P* = 0.020), 0.943 (*P* < 0.0001), 0.869 (*P* < 0.0001), 0.938 (*P* < 0.0001), 0.964 (*P* < 0.0001), 0.854 (*P* < 0.0001), 0.985 (*P* < 0.0001, 0.884 (*P* < 0.0001), for bladder urothelial carcinoma (BLCA), breast invasive carcinoma (BRCA), cervical cancer (CESC), bile duct cancer (CHOL), colon cancer (COAD), esophageal cancer (ESCA), glioblastoma (GBM), head and neck cancer (HNSC), kidney clear cell carcinoma (KIRC), kidney chromophobe (KICH), kidney papillary cell carcinoma (KIRP), liver cancer (LIHC), lung adenocarcinoma (LUAD), lung squamous cell carcinoma (LUSC), prostate cancer (PRAD), rectal cancer (READ), and stomach cancer (STAD). Because of the tissues derived from different organs, the diagnostic effects of PVT1 were also tested in the same organ cancers. PVT1 was a reliable diagnosis biomarkers for lung cancer, hepatobiliary cancer, renal tumor, colorectal carcinoma with the AUCs being 0.946 (0.924–0.969), 0.881 (0.849–0.913), 0.952 (0.938–0.967), 0.968 (0.934–0.999), respectively (Table 2).

**Table 2:** The differentiation and diagnosis value of PVT1 expression in cancers from different organs and corresponding normal ones of TCGA database

Series of Cancers	Type of tissue	NO. of Samples	Mann-Whitney *U* test		Diagnosis tese		
			*Z* value	*P* Value	AUC (95% CI)	Sensitivity	Specificity
lung cancer	cancer	1017	25.29	< 0.0001	0.946 (0.924–0.969)	0.895	0.902
	normal	112					
Uterine cancer	cancer	479	3.073	0.002	0.683 (0.588–0.777)	0.662	0.633
	normal	30					
Hepatobiliary Cancer	cancer	407	14.91	< 0.0001	0.881 (0.849–0.913)	0.83	0.82
	normal	61					
Renal tumor	cancer	889	34.46	< 0.0001	0.952 (0.938–0.967)	0.909	0.916
	normal	131					
Colorectal carcinoma	cancer	380	21.362	< 0.0001	0.968 (0.934–0.999)	0.979	0.944
	normal	54					

### Studies searching for PVT1 expression on the cancer diagnosis/ detection and quality assessment of diagnosis tests

The literature search resulted in 6 studies eligible for the meta-analysis (Supplementary Figure 1), and all were from China [11, 18–21, 23]. The studies involved 439 cancer patients and 434 controls, with mean sample size of 73.2 patients (range 20 to 111). Five different types of cancer were evaluated: gastric cancer (*n* = 2), clear cell renal cell carcinoma, melanoma, cervical cancer, and Non-small cell lung cancer (*n* = 1 each). The level of PVT1 was detected in patient’s tumor tissue or circulating blood by RT-PCR; and the negative control was adjacent noncancerous tissue or healthy serum. The main characteristics of each study are summarized in Table 3.

**Table 3:** Basic data for all included studies in the meta-analysis

Author	Year	Specimen	Derived	Cancer type	Diagnosis/detection tests			
					Case/control	AUC 95% CI	Sensitivity	Specificity
Wu Y, et al.	2016	blood	Fudan University Shanghai Cancer Center, China	clear cell renal cell carcinoma (ccRCC)	24/27	0.733 (0.582–0.884)	0.700	0.732
					37/35	0.682 (0.535–0.829)	0.644	0.624
Chen X, et al.	2017	blood	The253rd Hospital of PLA (Hohhot, Inner Mongolia, China).	Melanoma	51/47	0.939 (0.890–0.987)	0.941	0.851
Yang J, et al.	2016	blood	The Affiliated Hospital of Jiangxi University of Traditional Chinese Medicine	Cervical cancer(CC)	88/86	0.892 (0.838–0.946)	0.867	0.733
Gao J, et al.	2015	blood	Linyi People Hospital Hospital, Shandong Province, China	Gastric cancer(GC)	20/20	0.797 (0.731–0.863)	0.708	0.913
Yuan C, et al.	2015	Tissue	The First Affiliated Hospital of Nanjing Medical University and Yangzhou No.1 People’s hospital	Gastric cancer (GC)	111/111	0.728 (0.665–0.786)	0.802	0.604
Cui D, et al.	2015	Tissue	The First Affiliated Hospital of Nanjing Medical University	Non-small cell lung cancer (NSCLC).	108/108	0.736 (0.673–0.799)	0.815	0.617

Six published studies and 21 TCGA based diagnosis tests, with 8877 cases and 1290 controls, were enrolled. Each of them presented the AUC, sensitivity and specificity. In addition, the participants of a study were divided into two groups for testing and validation. Consequently, we assessed the overview quality of 28 diagnosis tests and reported them in Supplementary Figure 2. The risk of bias in patient selection was high in 28 tests (100%), mainly due to the 2-gate design (case-control) in the majority of tests. Because different test thresholds were selected to optimize sensitivity and specificity, the risk of bias of index test performance was high in 28 tests (100%). As some samples were deleted for the PVT1 expression undetected, the risk of bias arising from patient flow and timing of procedures was also considered high in the majority of studies (*n* = 23, 82%). However, the risk of bias for reference standard definition was low in the majority of studies (*n* = 28; 100%). Furthermore, for the regarding applicability, there was unclear risk identified for patient selection (*n* = 21, 75%), reference standard (*n* = 21, 75%), and low risk for reference standard (n = 28, 100%).

### Pooled effect of PVT1 on cancer diagnosis in tissue

**Figure 2:**
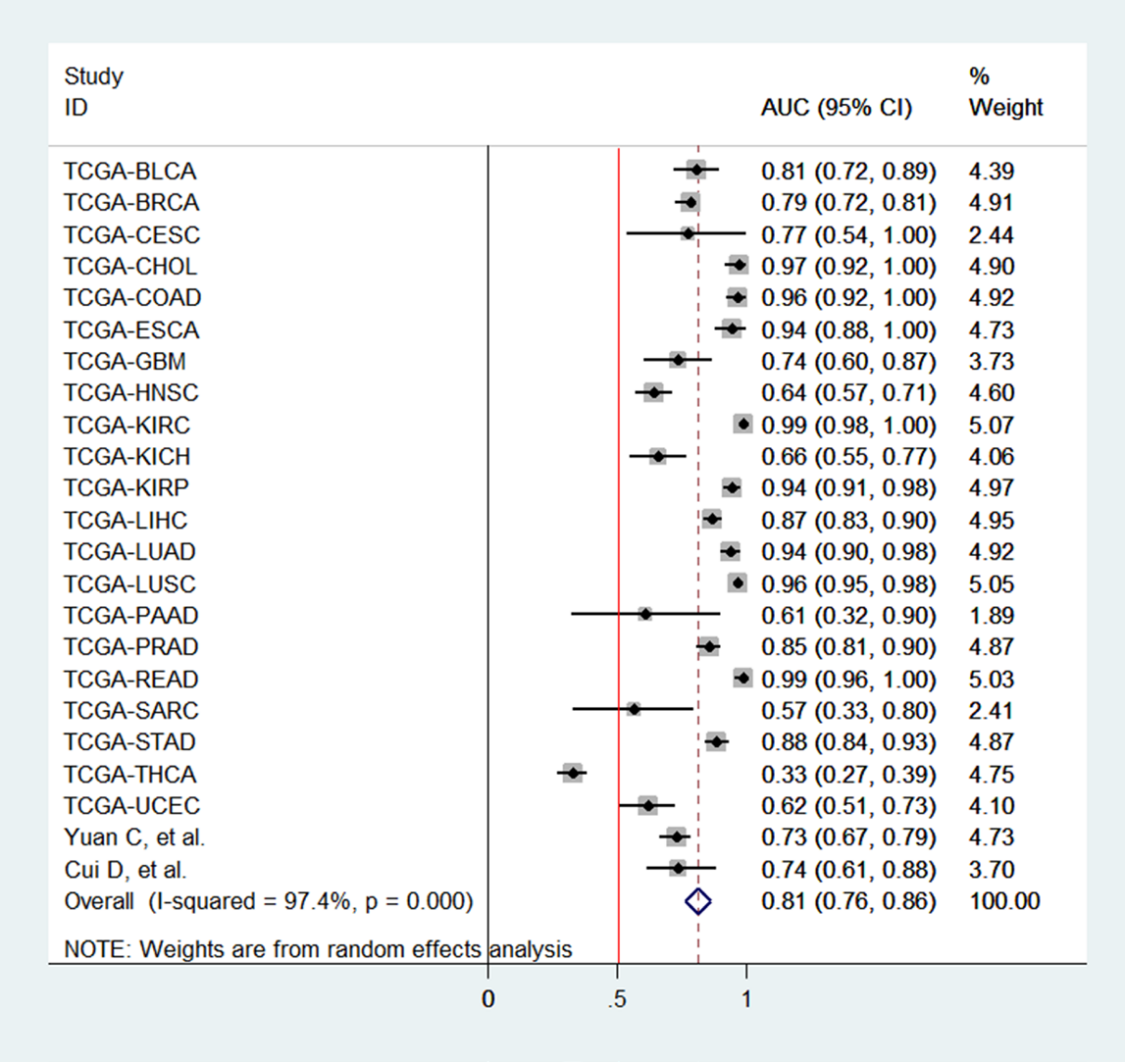
Pooled AUC of PVT1 for cancer diagnosis / detection in tissue Abbreviations: AUC, area under receiver operating characteristic curve; SE, standard error; IV, inverse variance methods; CI, confidence interval.

**Figure 3:**
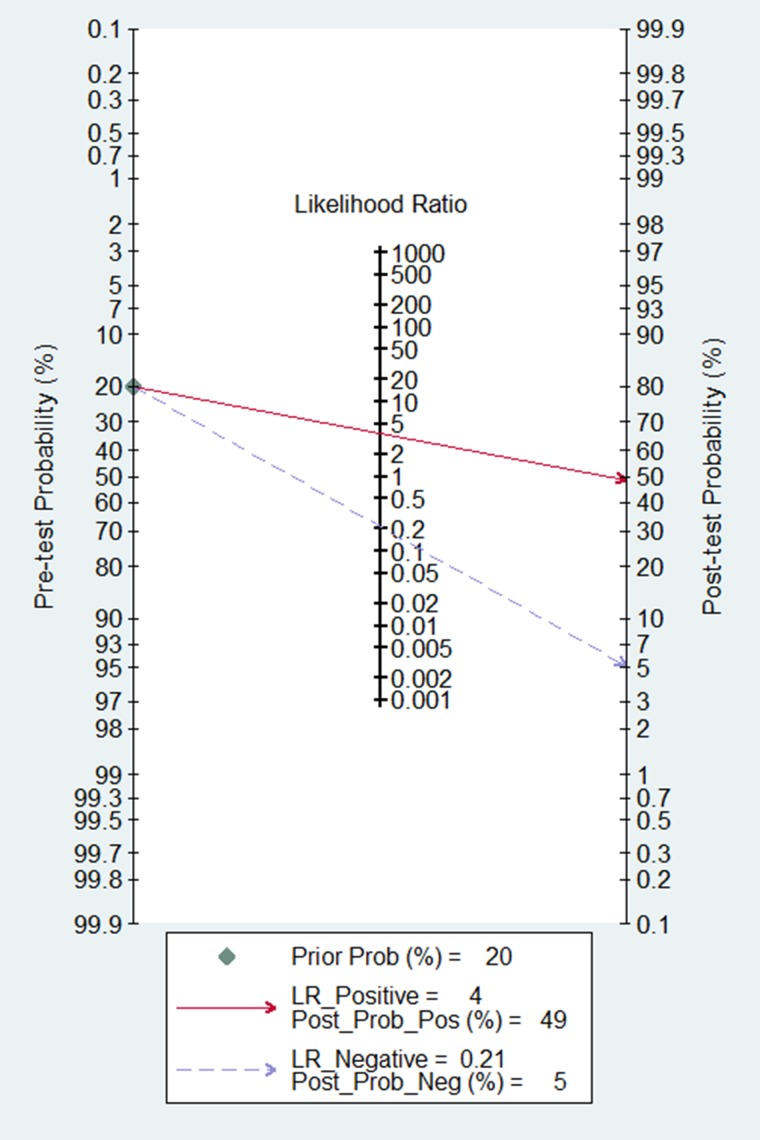
Fagan diagram evaluating the overall diagnostic value of PVT1 in tissue

**Figure 4:**
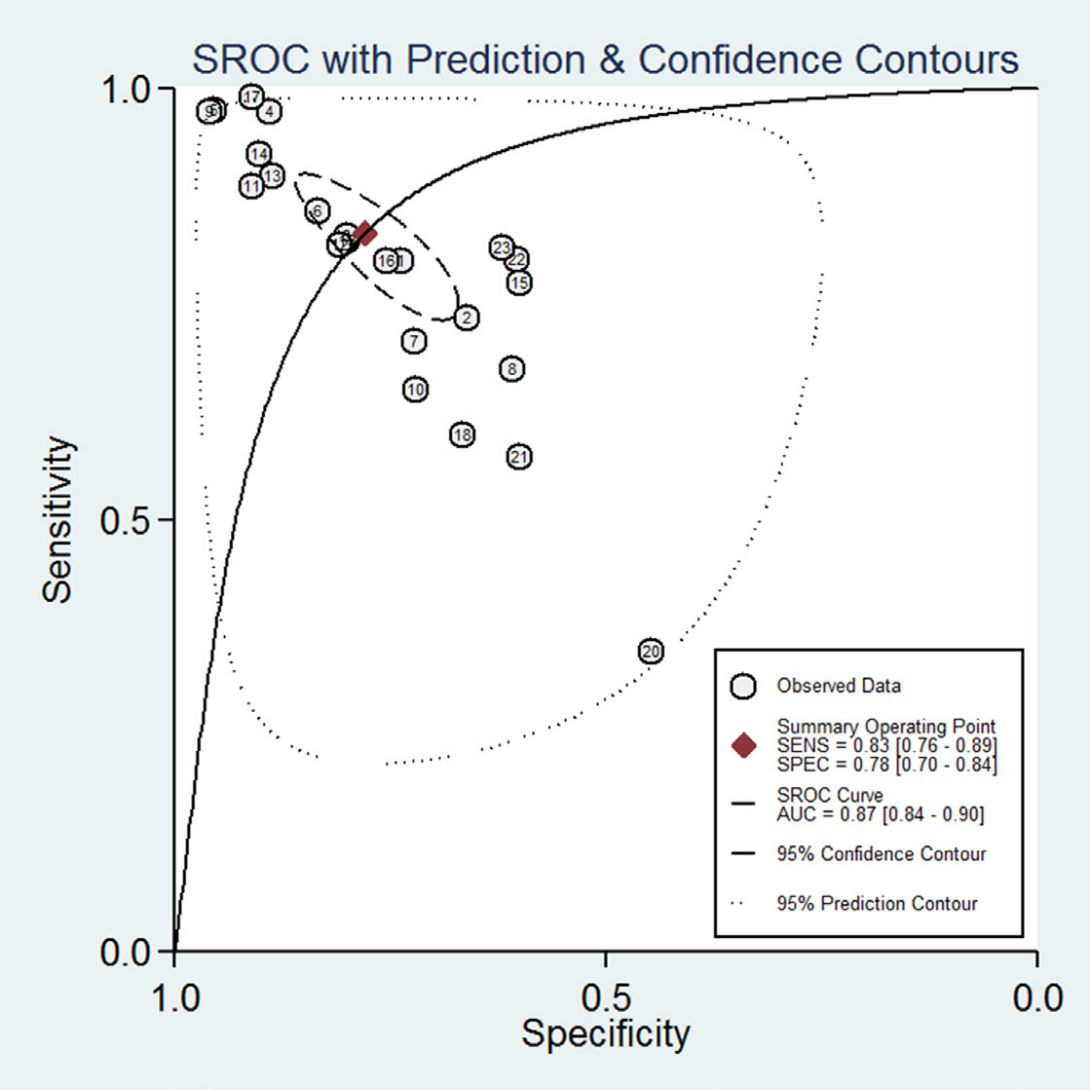
The SROC curve of PVT1 for the diagnosis of various cancers in tissue

According to the criteria of diagnosis tests performed in tissue, 2 published studies [11, 23] and 21 TCGA based diagnosis tests, with 8657 cancer tissues and 1075 corresponding normal ones, were chose. Because of severe heterogeneity resulting from severe threshold effect (spearman coefficient = −0.889, *P*_Q_ <0.0001) among 23 diagnosis tests (I^2^ = 97.4%, *P*_Q_ < 0.0001), the random-effects model was used to calculate the pooled effect. As shown in Figure 2, the pooled AUC was 0.81 (95% CI: 0.76–0.86). Furthermore, the pooled sensitivity, specificity, positive likelihood ratio (PLR), negative likelihood ratio (NLR), diagnostic odd ratio (DOR) was conducted with the values being 0.83 (95% CI: 0.76–0.89), 0.74 (95% CI: 0.70–0.84), 3.77 (95% CI: 2.56–5.54), 0.21 (95% CI: 0.14–0.34), 17.25 (95% CI: 8.43–35.27), respectively (Supplementary Figure 3). The Fagan plot and the summary operating characteristic curve (sROC) with AUC of 0.87 (95%CI: 0.84–0.90) were shown in Figure 3 and Figure 4; which suggested that the diagnostic accuracy of tissue PVT1 for cancers was relatively high.

### Pooled diagnostic values of circulating PVT1

**Figure 5:**
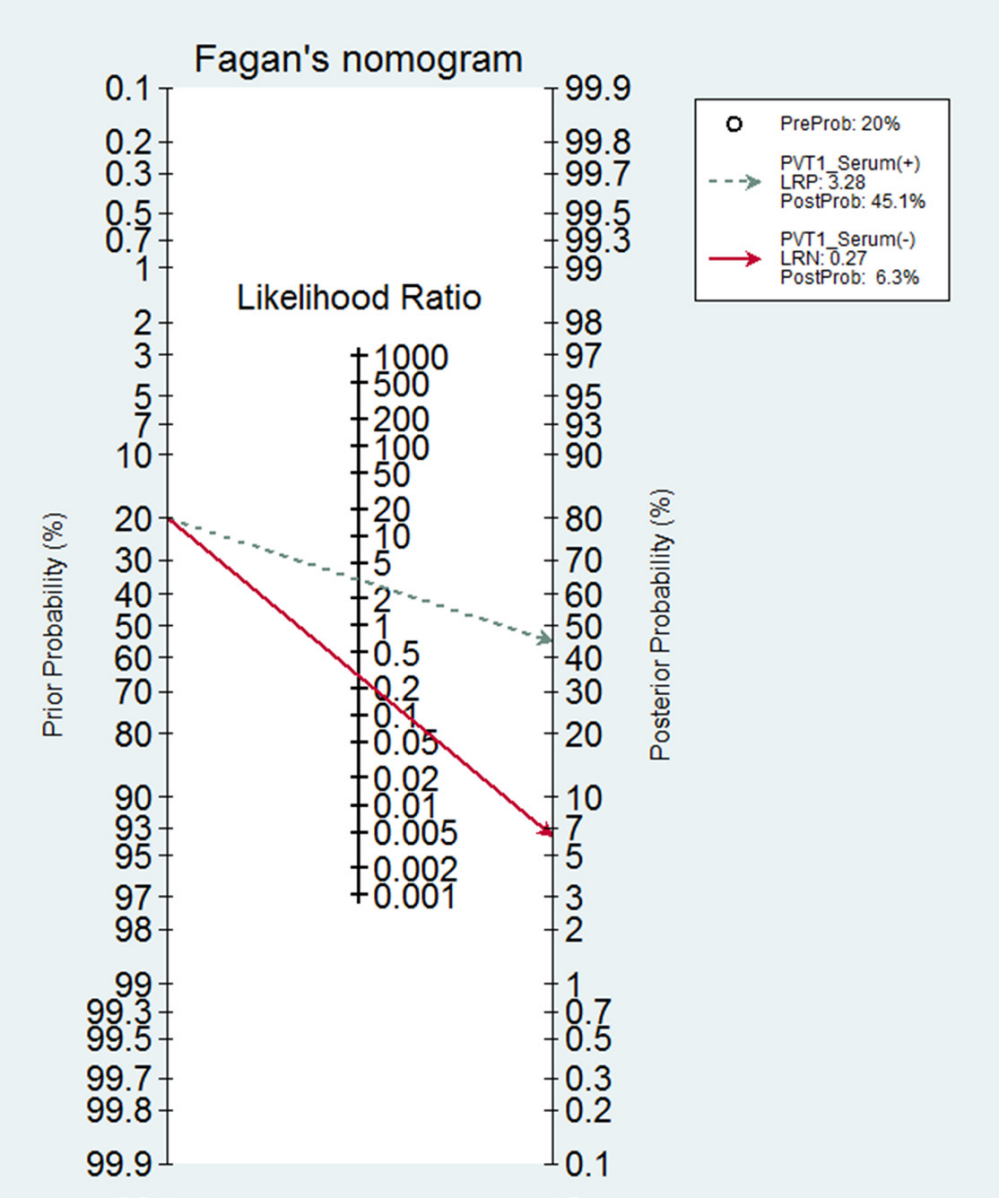
Fagan diagram evaluating the overall diagnostic value of circulating PVT1

**Figure 6:**
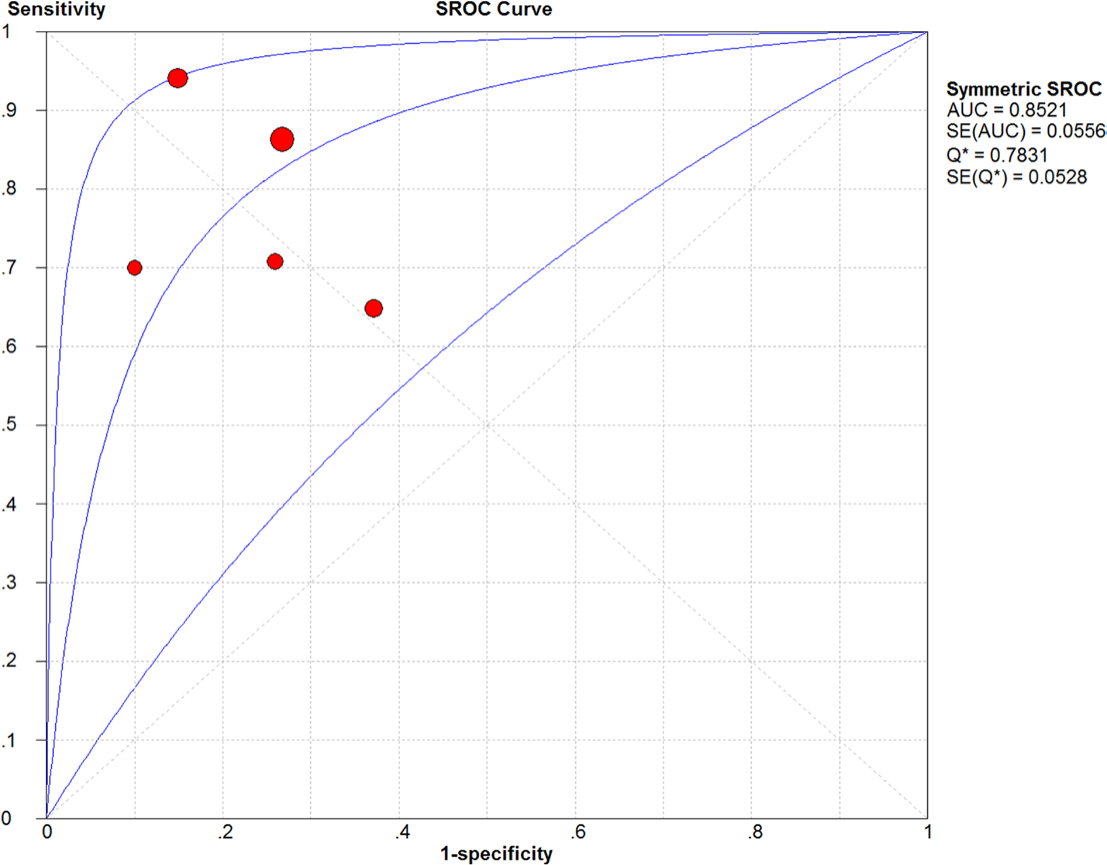
The SROC curve of circulating PVT1 for the diagnosis of various cancers

Four studies with 220 patients and 215 health controls showed data for circulating PVT1 on cancer detection/diagnosis. The pooled AUC was 0.83 (95%CI: 0.75–0.91) for 5 tests under the random-effects model (I^2^=82.1%, *P*_Q_ < 0.0001) (Supplementary Figure 4). With no significant threshold effect (spearman coefficient = -0.300, P_Q_ = 0.624), the pooled sensitivity, specificity, PLR, NLR, DOR were calculated to be 0.81 (95% CI: 0.76–0.86), 0.76 (95% CI: 0.70–0.81), 3.28 (95% CI: 2.05–5.25), 0.27 (95% CI: 0.14–0.51), 13.86 (95% CI: 4.70–40.66), respectively (Supplementary Figure 5). Moreover, the area under sROC was 0.85 (95%CI: 0.79–0.91). The diagnostic accuracy of circulating PVT1 on cancers was also relatively high with the Fagan plot and sROC curve present in Figure 5 and Figure 6.

### Sensitivity analysis

Sensitivity analysis was conducted for the association between cancer diagnosis/detection and PVT1 expression in tissues as well as in serum. Each diagnosis test was deleted in turn to examine the influence of the removed data on the overall AUC. The pooled AUC values of PVT1in tissue and serum remained above 0.50 throughout (data not shown), while the summary sensitivity and specificity, PLR, NLR, and area under sROC were altered (data not shown).

### Publication bias

**Figure 7:**
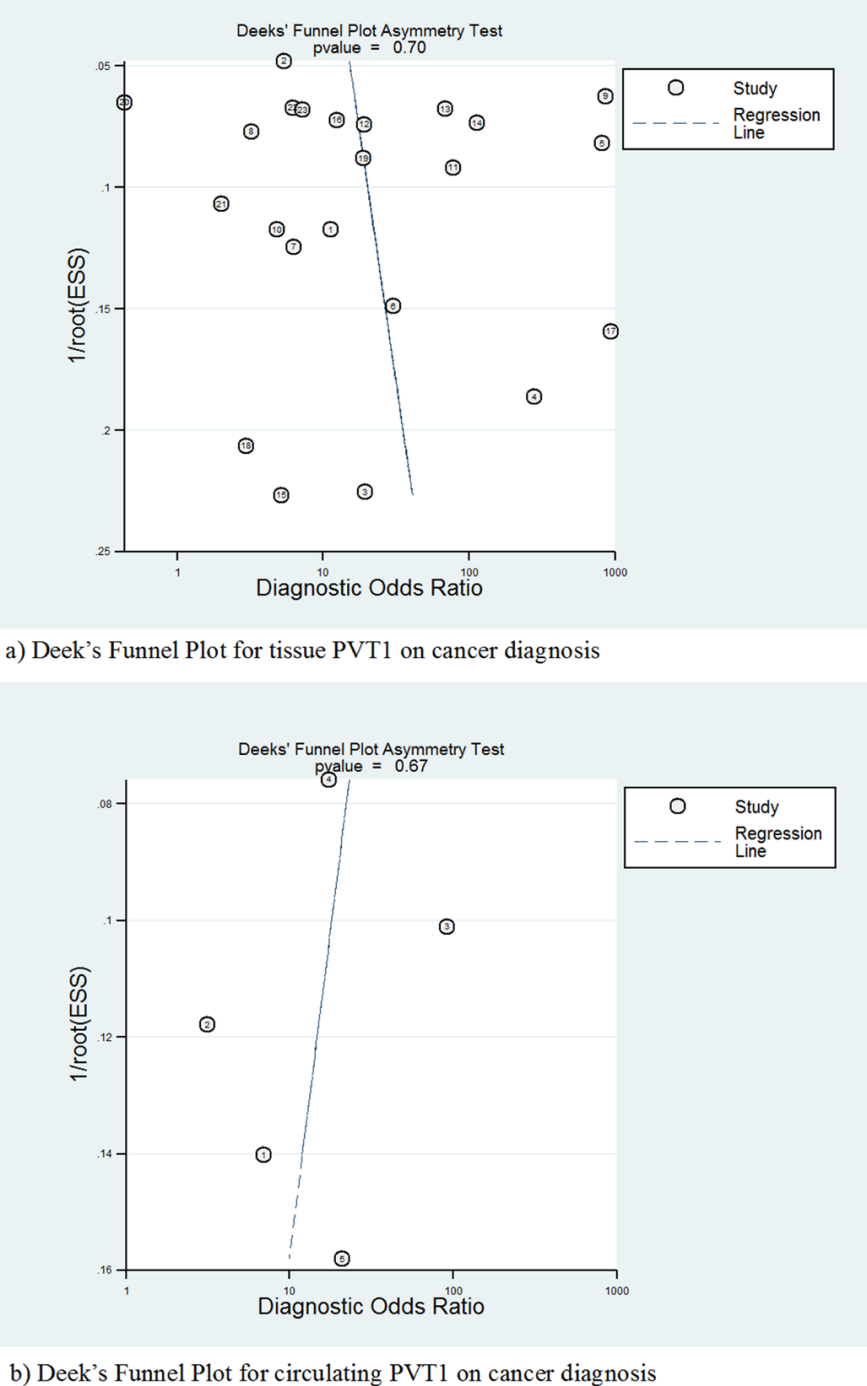
Deek’s funnel plot to evaluate the publication bias of test accuracy

Due to PVT1 expression acting as a diagnostic biomarker of cancer [24, 25], publication bias of test accuracy was checked by a Deek’s funnel plot (Figure 7), which showed that no significant bias existed in tissue (*t* = 0.39, *P* = 0.704) and serum (*t* = −0.47, *P* = 0.673).

## DISCUSSION

This current study aimed to analyze the differential expression of PVT1 in different types of common cancers and assess the effect of PVT1 expression on cancer diagnosis/detection. Basing on TCGA RNA-Seq data, the expression of PVT1 was suggested being a possible biomarker to distinguish cancer from normal tissue. The pooled effect showed that diagnostic accuracy of PVT1 for cancers was relatively high in tissue and serum. PVT1 might act as an effective biomarker for cancer diagnosis/detection.

With the advance on RNA-Seq technique and improvement of bioinformatics, numerous lncRNAs were detected and the representative RNA sequencing data of cancer was stored in TCGA database [26, 27], which provided more clues for cancer detection and therapy. However, only a few lncRNAs had been further explored to fully understand the role in development, diagnosis, and therapy of cancers. PVT1, a novel lncRNA initially found being co-expression with MYC, was confirmed that could promote the stability of MYC protein which participated in oncogene activation through Akt/c-Myc signaling pathway [10, 11, 28, 29]. In TCGA database, PVT1 could be detected in all included cancers. Our reanalysis of RNA-Seq data showed that PVT1 significantly upregulated in 18 types of cancerous tissues, as 16 could be accurately differentiated from corresponding normal tissue in the diagnosis tests. Furthermore, research in the mechanism found PVT1 could target genes such as LASP1 [34], FOXM1 [31], RSPO1 [32], p15, p16 [33], EZH2, TSHR [33], and NOP2 [35] to promote tumor cell proliferation, migration and invasive capability *in vitro*. Moreover, PVT1 could also contribute to the epithelial-to-mesenchymal transition (EMT), which was required for cancer metastasis and invasion [16, 36, 37]. Therefore, PVT1 is a common oncogenic lncRNA participating in tumor development and could be used as a biomarker for cancer detection / diagnosis.

In the present meta-analysis, the pooled-AUC of 0.81 (95% CI: 0.76–0.86) and the DOR of 17.25 (95% CI: 8.43–35.27) in tissues showed that the PVT1 had relatively high efficiency to distinguish cancer; although, the pooled sensitivity and specificity were not convincing for the significant threshold effect existing [38, 39]. Similar to the performance in tissues, the pooled–AUC of circulating PVT1 was still more than 0.80 with DOR being 13.86, which indicated that it was feasible to detect cancer by usingcirculating PVT1 [40, 41]. Meanwhile, the sensitivity of 0.83 (95% CI: 0.76–0.89) and the specificity of 0.74 (95% CI: 0.70–0.84) approved circulating PVT1 had a relatively high accuracy in human cancer detection. In addition, the Fagan’s nomogram showed circulating PVT1 could raise the probability of cancer detection by 25.1% (pos*t*-test probability 45.1% - pre-test probability 20%) [42], which was similar to effect practiced in tissue. The pooled diagnostic values of circulating PVT1, like H19 [43], HULC [44], miR-31 [30], was higher than that of traditional clinical markers such as CEA and CA19–9. It all suggested that PVT1 expression, especially in serum, was a higher effective biomarker for human cancer detection.

Some meta-analyses focused on the association of lncRNAs such as BANCR [45], HOTTIP [46], CCAT2 [47], and metastasis as well as prognosis of cancers; all of them were based on the lncRNAs detected in tissues. To search for an applicable diagnosis biomarker, we focused on the effect of PVT1 expression, especially in serum, on diagnosis / detection. To our best knowledge, this is the first meta-analysis of PVT1 expression on cancer detection with the data from TCGA and published studies.

Our study contains some limitations. First, the samples of controls were few and publication bias existed. Second, because of severe threshold effect in TCGA data based analysis; the diagnostic accuracy of PVT1 could not be accurately confirmed in tissue. Third, because of the nature of the meta-analysis using aggregated group data, the confounding factors could not be controlled. Fourth, there were few studies on association of serum PVT1 expression with cancer diagnosis / detection, some of our significant findings was limited by the low precision as indicated by the wide confidence intervals. Therefore, studies with larger-scale, multicenter, high-quality and referring to multi-type cancer are needed to confirm our findings.

## MATERIALS AND METHODS

### TCGA sequencing data

PVT1 RNA sequencing datasets of different cancers and corresponding normal tissues were downloaded from https://xenabrowser.net/heatmap/ (TCGA database) with the format being Illumina Hiseq Pancan normalized, when the relative clinical data was from https://portal.gdc.cancer.gov/projects/(TCGA database).

### Literature search strategy

Reports of studies in English or Chinese language on the role of PVT1 in human cancer were searched in PubMed, EMBASE, Cochrane Library, China National Knowledge Infrastructure, and Wanfang databases with the keywords “PVT1 and (cancer or tumor or neoplasm)”. References of retrieved papers and conference reports were also searched to identify relevant studies. The last searching date was May 8, 2017.

### Selection criteria of reported research

The titles and abstracts of searched articles were checked by 3 authors (YZ, TW, ZS) after duplicates removed. Then, the full text of eligible articles was retrieved. Eligible articles should have the following criteria: 1) the expression of PVT1 was analyzed by detection/diagnosis of human cancer, 2) the expression of PVT1 was tested in cancer tissue or circulating blood by RT-PCR, fluorescence *in-situ* hybridization or RNA-Seq, and 3) diagnostic test indexes for detection/diagnosis (sensitivity, specificity, and AUC) were provided or could be calculated from the available data. Studies not fulfilling the criteria, reviews, animal/cell-line studies, and case reports were excluded. Furthermore, if more than 1 report from the same cohort was published, only the most recent publication was included. Consensus in searching and exclusion was resolved by discussion and with other 2 investigators (XC, DH) if needed.

### Data extraction and quality assessment

Two authors (YL, PL) extracted the following data by using an extraction form: first author’s name, published year, region of cohort, sample size, cancer type, method to test PVT1, AUC, sensitivity, and specificity. The quality of diagnostic test studies was assessed by the Quality Assessment of Diagnostic Accuracy Studies 2 (QUADAS2).

### Statistical methods

Mann-Whitney *U* test was applied to analyze the differential expression of PVT1between cancerous tissues and corresponding normal ones. ROC curve was performed to assess the effect of PVT1 expression in cancer diagnosis/detection. In the meta-analysis, the heterogeneity among studies was tested by Inconsistency (I^2^) and Q tests (chi-square test). If no statistical heterogeneity was found (I^2^ < 50%, *P*_Q_ > 0.05), a fixed-effects model was used to estimate the pooled sensitivity, specificity, positive likelihood ratio (PLR), negative likelihood ratio (NLR), diagnostic odd ratio (DOR), and summary operating characteristic curve (sROC). Otherwise, a random-effects model was used. Moreover, Deek’s tests were used to assess publication bias. In addition, Engauge Digitizer 4.1 and Origin 8 were used to analyze AUC, when AUC and 95% CIs were not provided directly in some studies. All tests, being considered statistically significant with *P* < 0.05, were two sided and performed by STATA 14.0, Meta-DiSc 1.4, and Review Manager 5.3 (Cochrane network).

## CONCLUSIONS

This meta-analysis is the first to demonstrate that high expression of the long noncoding RNA PVT1 is related to cancer detection. The expression of PVT1, especially tested in serum, might be a biomarker for cancer diagnosis / detection.

### Ethical approval

This article does not contain any studies with human participants or animals performed by any of the authors.

### Informed consent

Informed consent was obtained from all individual participants included in the study.

### Consent for publication

Not applicable.

## SUPPLEMENTARY MATERIALS FIGURES



## Abbreviations

lncRNA: long noncoding RNA
RNA-Seq: high-throughput RNA sequencing
TCGA: the Cancer Genome Atlas
AUC: area under receiver operating characteristic curve
PLR, NLR: positive likelihood ratio, negative likelihood ratio
DOR: diagnostic odd ratio
sROC: summary operating characteristic curve
EMT: epithelial-to-mesenchymal transition
CEA: carcinoembryonic antigen
CA: carbohydrate antigen
PRISM: Preferred Reporting Items for Systematic Reviews and Meta-Analyses
CNKI: China National Knowledge Infrastructure
TP: true positives
FP: false positives
FN: false negatives
TN: true negatives
QUADAS2: Quality Assessment of Diagnostic Accuracy Studies 2
