# Supplementary material for: LncRNA PVT1 as an effective biomarker for cancer diagnosis and detection based on transcriptome data and meta-analysis
Source: Oncotarget. 2017 Sep 4;8(43):75455–66. doi: 10.18632/oncotarget.20634 (PMC5650436; doi:10.18632/oncotarget.20634)
Supplement: Supplementary file 1 [file oncotarget-08-75455-s001.pdf]

LncRNA PVT1 as an effective biomarker for cancer diagnosis and detection based on transcriptome data and meta-analysis

SUPPLEMENTARY MATERIALS

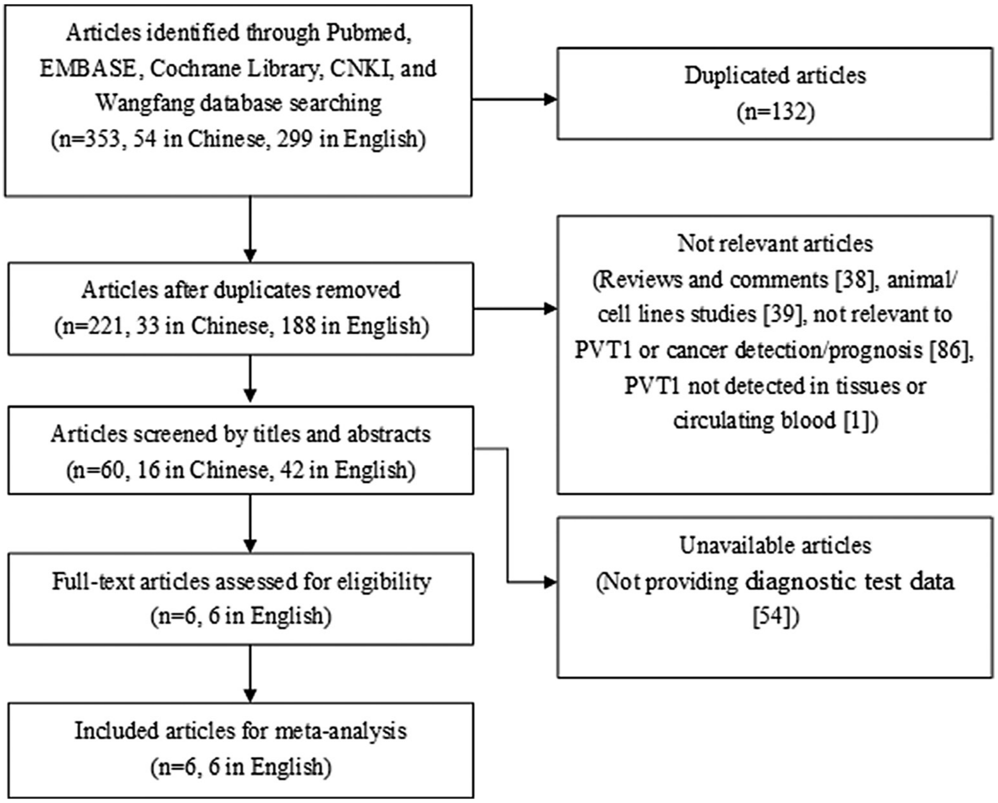

Supplementary Figure 1: The flow chart of meta-analysis.

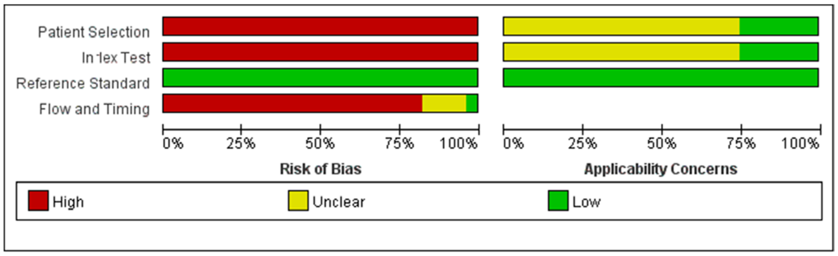

Methodological quality graph

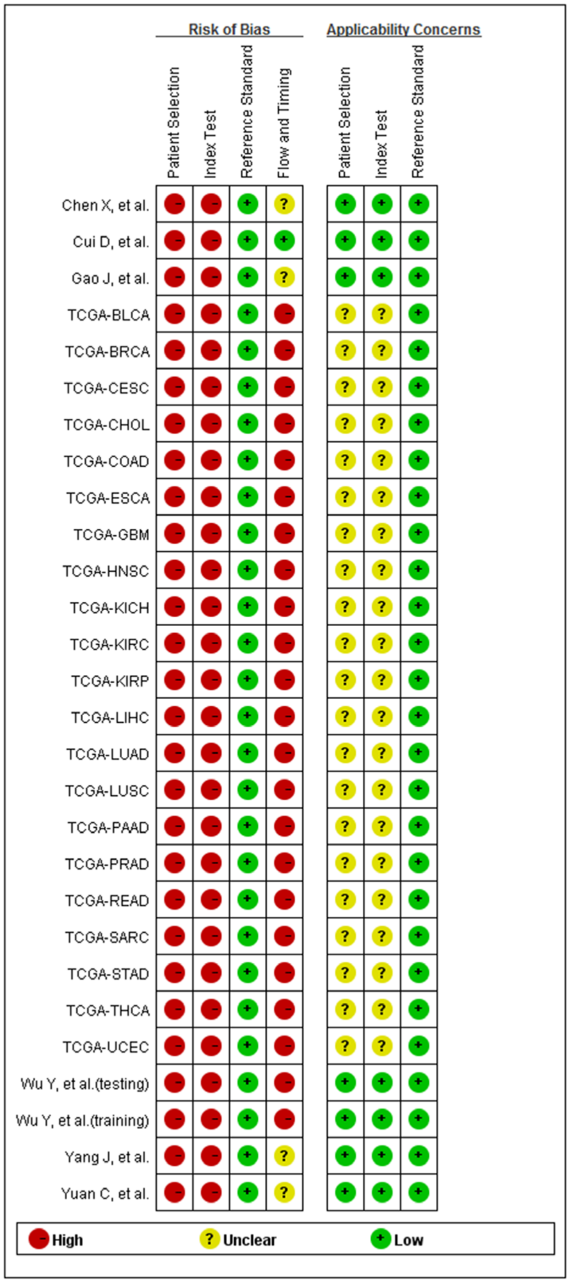

Methodological quality summary

Supplementary Figure 2: Methodological quality of PVT1 for the diagnosis of various cancers.

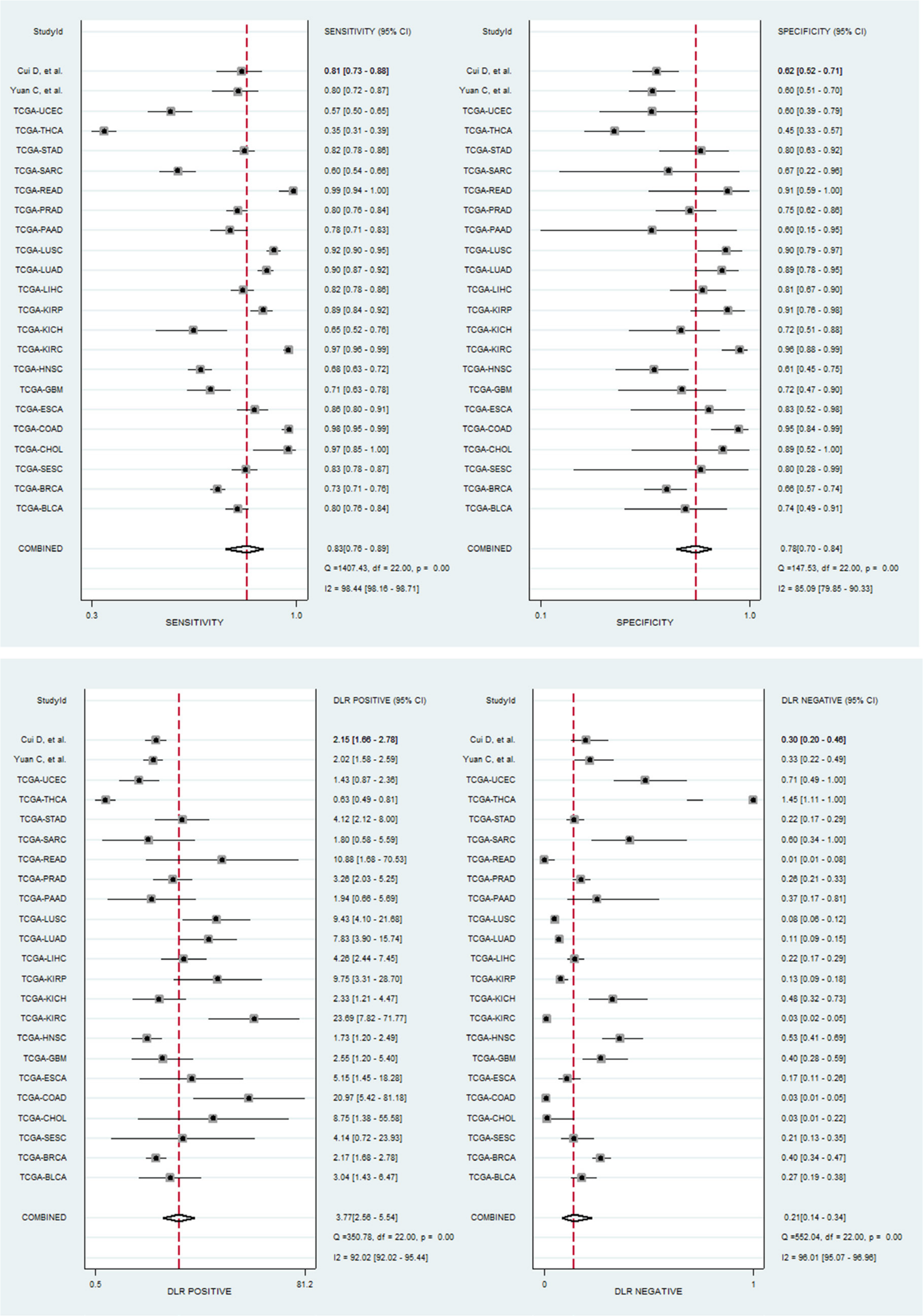
StudyId
SENSITIVITY (95% CI)
Cui D, et al. 0.81 [0.73 - 0.88]
Yuan C, et al. 0.80 [0.72 - 0.87]
TCGA-UCEC 0.57 [0.50 - 0.65]
TCGA-THCA 0.35 [0.31 - 0.39]
TCGA-STAD 0.82 [0.78 - 0.86]
TCGA-SARC 0.60 [0.54 - 0.66]
TCGA-READ 0.99 [0.94 - 1.00]
TCGA-PRAD 0.80 [0.76 - 0.84]
TCGA-PAAD 0.78 [0.71 - 0.83]
TCGA-LUSC 0.92 [0.90 - 0.95]
TCGA-LUAD 0.90 [0.87 - 0.92]
TCGA-LIHC 0.82 [0.78 - 0.86]
TCGA-KIRP 0.89 [0.84 - 0.92]
TCGA-KICH 0.65 [0.52 - 0.76]
TCGA-KIRC 0.97 [0.96 - 0.99]
TCGA-HNSC 0.68 [0.63 - 0.72]
TCGA-GBM 0.71 [0.63 - 0.78]
TCGA-ESCA 0.86 [0.80 - 0.91]
TCGA-COAD 0.98 [0.95 - 0.99]
TCGA-CHOL 0.97 [0.85 - 1.00]
TCGA-SESC 0.83 [0.78 - 0.87]
TCGA-BRCA 0.73 [0.71 - 0.76]
TCGA-BLCA 0.80 [0.76 - 0.84]
COMBINED 0.83[0.76 - 0.89]
Q =1407.43, df = 22.00, p = 0.00
I2 = 98.44 [98.16 - 98.71]
0.3
SENSITIVITY
1.0
StudyId
SPECIFICITY (95% CI)
Cui D, et al. 0.62 [0.52 - 0.71]
Yuan C, et al. 0.60 [0.51 - 0.70]
TCGA-UCEC 0.60 [0.39 - 0.79]
TCGA-THCA 0.45 [0.33 - 0.57]
TCGA-STAD 0.80 [0.63 - 0.92]
TCGA-SARC 0.67 [0.22 - 0.96]
TCGA-READ 0.91 [0.59 - 1.00]
TCGA-PRAD 0.75 [0.62 - 0.86]
TCGA-PAAD 0.60 [0.15 - 0.95]
TCGA-LUSC 0.90 [0.79 - 0.97]
TCGA-LUAD 0.89 [0.78 - 0.95]
TCGA-LIHC 0.81 [0.67 - 0.90]
TCGA-KIRP 0.91 [0.76 - 0.98]
TCGA-KICH 0.72 [0.51 - 0.88]
TCGA-KIRC 0.96 [0.88 - 0.99]
TCGA-HNSC 0.61 [0.45 - 0.75]
TCGA-GBM 0.72 [0.47 - 0.90]
TCGA-ESCA 0.83 [0.52 - 0.98]
TCGA-COAD 0.95 [0.84 - 0.99]
TCGA-CHOL 0.89 [0.52 - 1.00]
TCGA-SESC 0.80 [0.28 - 0.99]
TCGA-BRCA 0.66 [0.57 - 0.74]
TCGA-BLCA 0.74 [0.49 - 0.91]
COMBINED 0.78[0.70 - 0.84]
Q =147.53, df = 22.00, p = 0.00
I2 = 85.09 [79.85 - 90.33]
0.1
SPECIFICITY
1.0
StudyId
DLR POSITIVE (95% CI)
Cui D, et al. 2.15 [1.66 - 2.78]
Yuan C, et al. 2.02 [1.58 - 2.59]
TCGA-UCEC 1.43 [0.87 - 2.36]
TCGA-THCA 0.63 [0.49 - 0.81]
TCGA-STAD 4.12 [2.12 - 8.00]
TCGA-SARC 1.80 [0.58 - 5.59]
TCGA-READ 10.88 [1.68 - 70.53]
TCGA-PRAD 3.26 [2.03 - 5.25]
TCGA-PAAD 1.94 [0.66 - 5.69]
TCGA-LUSC 9.43 [4.10 - 21.68]
TCGA-LUAD 7.83 [3.90 - 15.74]
TCGA-LIHC 4.26 [2.44 - 7.45]
TCGA-KIRP 9.75 [3.31 - 28.70]
TCGA-KICH 2.33 [1.21 - 4.47]
TCGA-KIRC 23.69 [7.82 - 71.77]
TCGA-HNSC 1.73 [1.20 - 2.49]
TCGA-GBM 2.55 [1.20 - 5.40]
TCGA-ESCA 5.15 [1.45 - 18.28]
TCGA-COAD 20.97 [5.42 - 81.18]
TCGA-CHOL 8.75 [1.38 - 55.58]
TCGA-SESC 4.14 [0.72 - 23.93]
TCGA-BRCA 2.17 [1.68 - 2.78]
TCGA-BLCA 3.04 [1.43 - 6.47]
COMBINED 3.77[2.56 - 5.54]
Q =350.78, df = 22.00, p = 0.00
I2 = 92.02 [92.02 - 95.44]
0.5
DLR POSITIVE
81.2
StudyId
DLR NEGATIVE (95% CI)
Cui D, et al. 0.30 [0.20 - 0.46]
Yuan C, et al. 0.33 [0.22 - 0.49]
TCGA-UCEC 0.71 [0.49 - 1.00]
TCGA-THCA 1.45 [1.11 - 1.00]
TCGA-STAD 0.22 [0.17 - 0.29]
TCGA-SARC 0.60 [0.34 - 1.00]
TCGA-READ 0.01 [0.01 - 0.08]
TCGA-PRAD 0.26 [0.21 - 0.33]
TCGA-PAAD 0.37 [0.17 - 0.81]
TCGA-LUSC 0.08 [0.06 - 0.12]
TCGA-LUAD 0.11 [0.09 - 0.15]
TCGA-LIHC 0.22 [0.17 - 0.29]
TCGA-KIRP 0.13 [0.09 - 0.18]
TCGA-KICH 0.48 [0.32 - 0.73]
TCGA-KIRC 0.03 [0.02 - 0.05]
TCGA-HNSC 0.53 [0.41 - 0.69]
TCGA-GBM 0.40 [0.28 - 0.59]
TCGA-ESCA 0.17 [0.11 - 0.26]
TCGA-COAD 0.03 [0.01 - 0.05]
TCGA-CHOL 0.03 [0.01 - 0.22]
TCGA-SESC 0.21 [0.13 - 0.35]
TCGA-BRCA 0.40 [0.34 - 0.47]
TCGA-BLCA 0.27 [0.19 - 0.38]
COMBINED 0.21[0.14 - 0.34]
Q =552.04, df = 22.00, p = 0.00
I2 = 96.01 [95.07 - 96.96]
0
DLR NEGATIVE
1

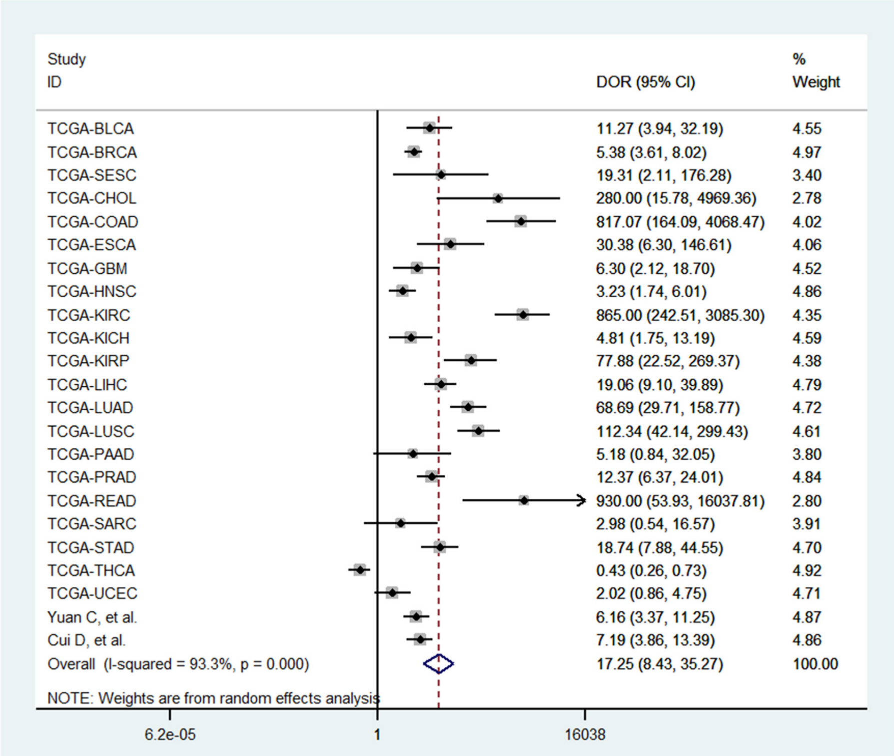

Supplementary Figure 3: The pooled effect value of PVT1 in 23 diagnosis tests of tissue.

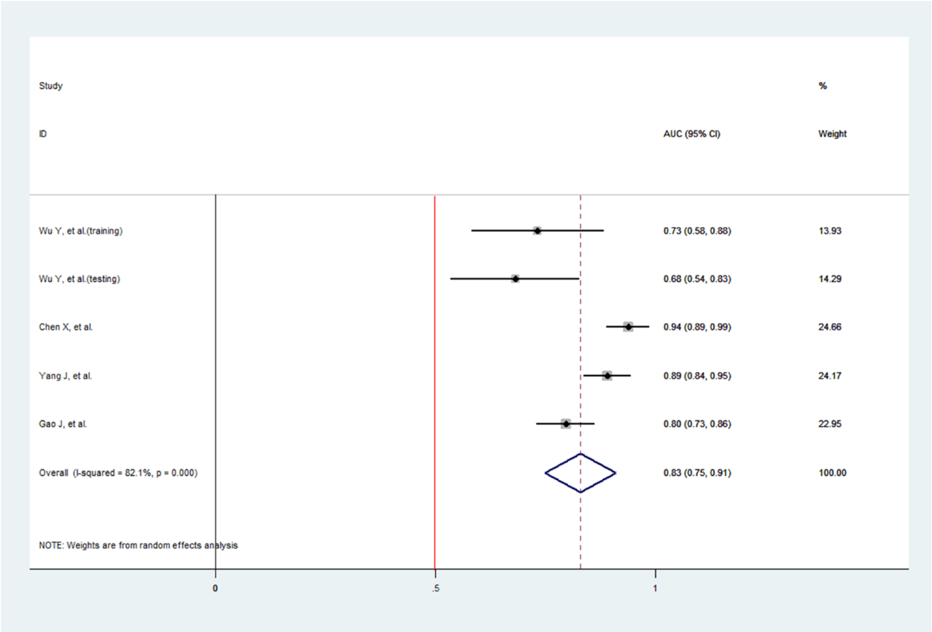

Supplementary Figure 4: Pooled AUC of circulating PVT1 for cancer diagnosis/detection.

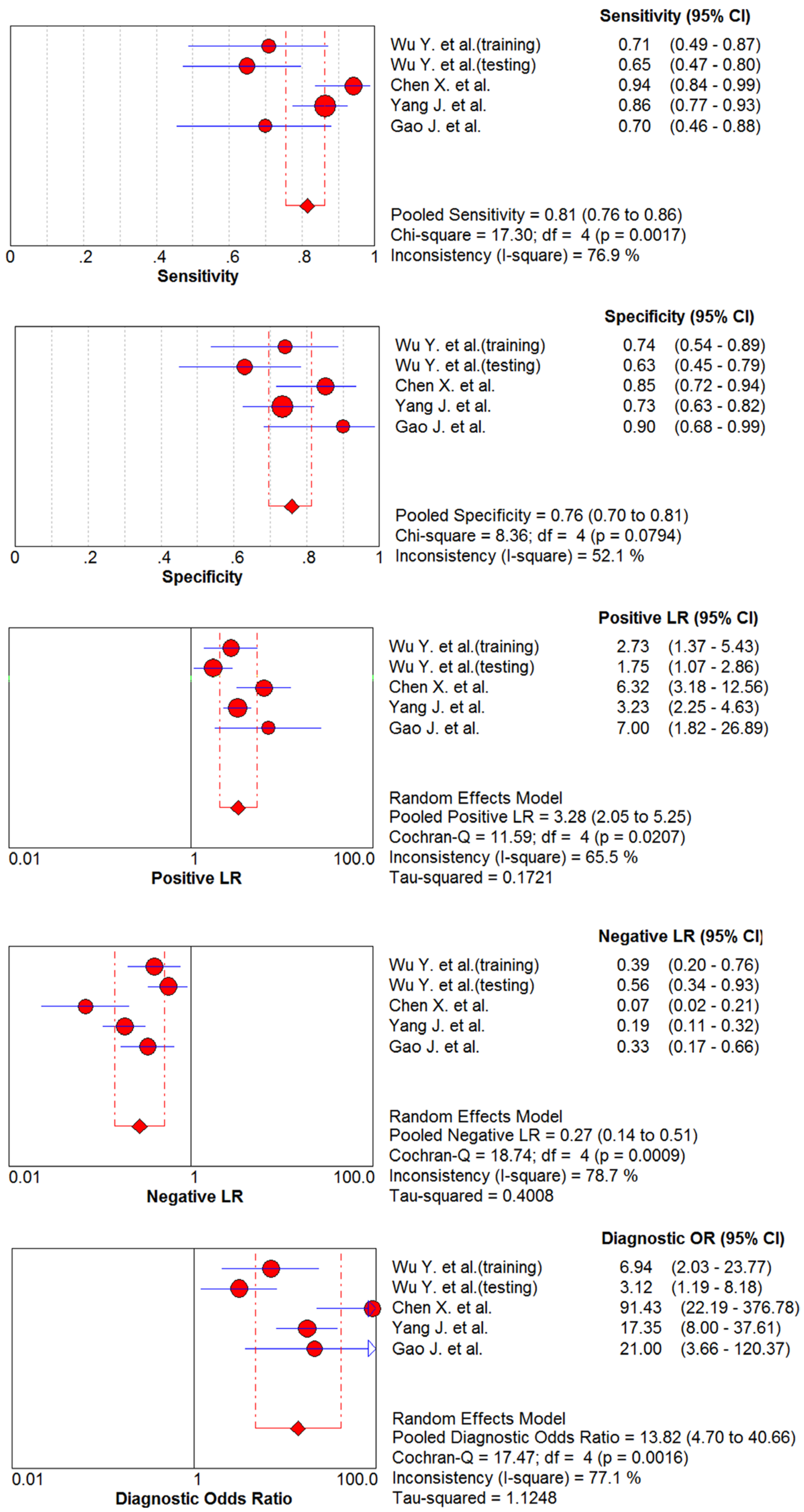

Supplementary Figure 5: The pooled effect value of circulating PVT1 in 5 diagnosis tests.
